# Supplementary material for: In Vitro Cell Interactions on PVDF Films: Effects of Surface Morphology and Polar Phase Transition
Source: Materials (Basel). 2021 Sep 11;14(18):5232. doi: 10.3390/ma14185232 (PMC8470707; doi:10.3390/ma14185232)
Supplement: Supplementary file 1 [file materials-14-05232-s001.zip › materials-1313464-supplementary.pdf]

Supplementary data

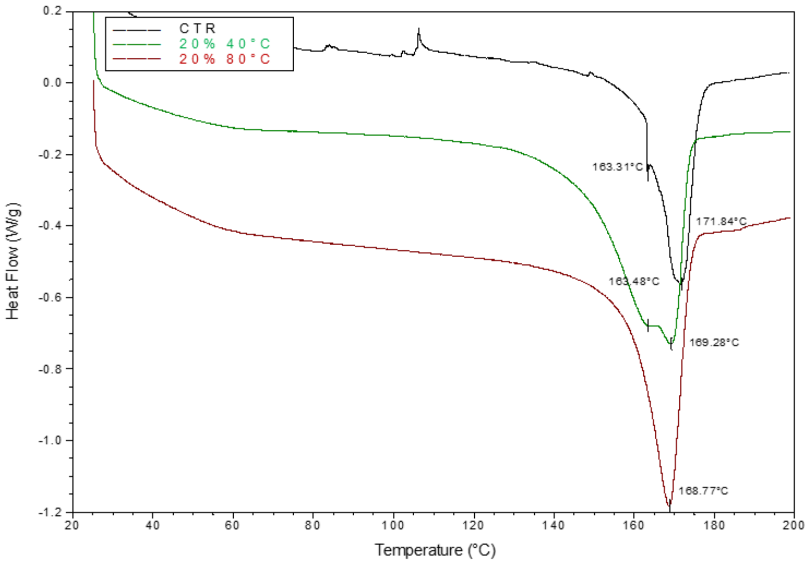

Figure S1. DSC thermograms of samples (20%) casted at different temperatures (40 °C, 80 °C). Neat PVDF pellets were used as control (CTR).

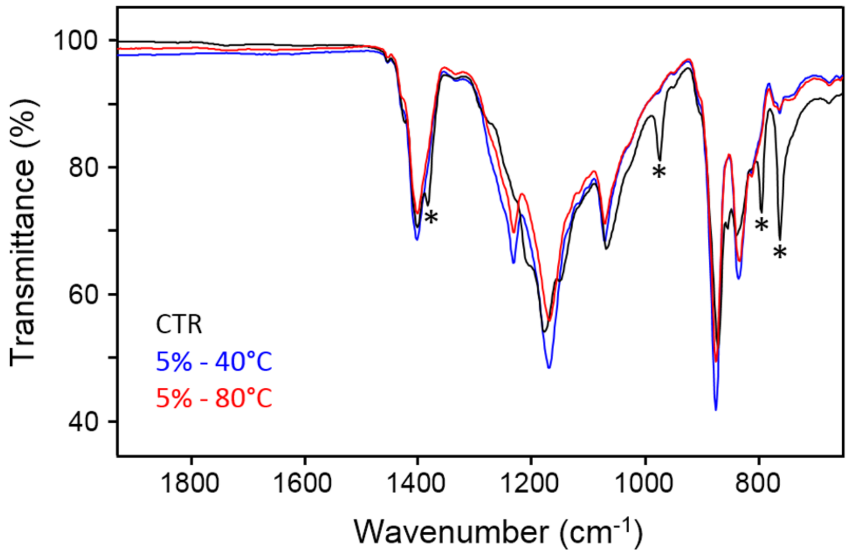

Figure S2. PVDF films cast from 5% solution under different temperature conditions (40 °C, 80 °C). Asterisks denote peaks characteristic of the phase.
